# CgOpt1, a putative oligopeptide transporter from *Colletotrichum gloeosporioides *that is involved in responses to auxin and pathogenicity

**DOI:** 10.1186/1471-2180-9-173

**Published:** 2009-08-21

**Authors:** Véronique Chagué, Rudy Maor, Amir Sharon

**Affiliations:** 1 Department of Plant Sciences, Tel Aviv University, Tel Aviv 69978, Israel; 2 Current address: Rosetta Genomics, 10 Plaut Street, Rehovot, 76706, Israel

## Abstract

**Background:**

The fungus *Colletotrichum gloeosporioides *f. sp. *aeschynomene *produces high levels of indole-3-acetic acid (IAA) in axenic cultures and during plant infection. We generated a suppression subtractive hybridization library enriched for IAA-induced genes and identified a clone, which was highly expressed in IAA-containing medium.

**Results:**

The corresponding gene showed similarity to oligopeptide transporters of the OPT family and was therefore named *CgOPT1*. Expression of *CgOPT1 *in mycelia was low, and was enhanced by external application of IAA. *cgopt1*-silenced mutants produced less spores, had reduced pigmentation, and were less pathogenic to plants than the wild-type strain. IAA enhanced spore formation and caused changes in colony morphology in the wild-type strain, but had no effect on spore formation or colony morphology of the *cgopt1*-silenced mutants.

**Conclusion:**

Our results show that IAA induces developmental changes in *C. gloeosporioides*. These changes are blocked in *cgopt1*-silenced mutants, suggesting that this protein is involved in regulation of fungal response to IAA. *CgOPT1 *is also necessary for full virulence, but it is unclear whether this phenotype is related to auxin.

## Background

Fungi can produce plant hormones in axenic cultures when supplemented with the appropriate precursors [1]. For production of the hormone indole-3-acetic acid (IAA), tryptophan must be supplied: no IAA is produced without external tryptophan, and the amount of IAA increases with increasing tryptophan concentrations [1-5].

Various effects of IAA on fungi have been reported. IAA and gibberellic acid were reported to affect yeast sporulation and cell elongation, but the effects of IAA were not uniform and varied according to growth conditions, such as vitamin content in the culture medium [6]. IAA also induced invasive growth in *Saccharomyces cerevisiae*, suggesting that it activates the pheromone MAP kinase pathway [7]. In *Neurospora crassa*, IAA reduced the 'spore density effect' and germination occurred at high densities in the presence of auxin [8]. In *Aspergillus nidulans*, IAA partially restored cleistothecium formation and fertility of a tryptophan-auxotrophic strain [9]. External application of IAA has been shown to have various effects in additional fungal species, but it has been difficult to determine whether the observed phenotypes represent the physiological effects of endogenous fungal IAA [1,10].

The possible role of fungal IAA in plant diseases is also ambiguous. Auxin compounds produced by antagonistic and pathogenic *Pythium *spp. were shown to stimulate plant growth [11]. Pre-treatment of potato tubers at the inoculation sites attenuated the extent of *Fusarium eumartii *damage and was correlated with a decrease in several of the fungu's extracellular hydrolytic activities [12]. An IAA-overproducing strain of the mycorrhizal fungus *Hebeloma cylindrosporum *had a more pronounced impact on *Pinus pinaster *cortical cell elongation and radial diameter than the wild-type strain [13]. It should be noted that in that study IAA production was determined under culture conditions in the presence of high tryptophan concentrations and *in-planta *production of IAA by the mycorrhizal fungus was not verified. IAA-overproducing *Fusarium *strains were generated by expressing the bacterial *iaaM *and *iaaH *genes in two species pathogenic to *Orobanche *[14]. The transgenic strains produced more IAA in culture and demonstrated enhanced virulence on the host plants. Again, *in-planta *production of IAA was not determined.

Most fungi produce IAA from the amino acid tryptophan through the indole-3-pyruvic acid (IPY) pathway [1]. Genes of the IPY pathway have been recently identified in the smut fungus *Ustilago maydis *[15]. Two indole-3-acetaldehyde dehydrogenase genes (*IAD1, IAD2*) were identified and *Δiad1Δiad2 *mutant strains were produced. These mutants were blocked in the conversion of both indole-3-acetaldehyde and tryptamine to IAA. Furthermore, deletion of two aromatic amino acid aminotransferases (*TAM1 *and *TAM2*, required for conversion of tryptophan to IPY) in the *Δiad1Δiad2 *mutant background resulted in a further decrease in IAA production. IAA levels were reduced in plants infected with the mutant strains compared to wild-type infected plants, but tumor formation was unaffected. Thus, although these results strongly suggest that *U. maydis *produces IAA within the plant, they do not provide answers as to the possible role or effect of fungus-produced IAA on disease development.

We previously showed that *Colletotrichum gloeosporioides *f. sp. *aeschynomene *(*C. gloeosporioides*) produces large quantities of IAA in axenic culture [16]. Unlike in other fungi, the major IAA-biosynthesis pathway in *C. gloeosporioides *is the bacterial indole-3-acetamide (IAM) pathway. Although external addition of tryptophan was necessary for the production of IAA in axenic cultures, *in-planta *production of IAA by the fungus was also demonstrated [17]. To gain insight into the possible roles of IAA, we developed a screen for auxin-induced genes in *C. gloeosporioides*. Here we report the identification and characterization of *CgOPT1*, a *C. gloeosporioides *IAA-responsive gene, which is involved in mediating fungal responses to IAA.

## Results

### Isolation and characterization of *CgOPT1*

In search of IAA-induced fungal genes, a suppressive subtraction hybridization (SSH) library was prepared from mycelia grown in media with (+) or without (-) IAA. Putative differential clones were sequenced and analyzed by BlastX and their expression was verified by northern blot analysis (data not shown). Only three clones showed consistent induction by IAA: Cas2 (accession no. FJ014488), which showed homology to an integral membrane protein (92% similarity and 72% identity to *Aspergillus clavatus *EAW10960.1), Cas51, which showed homology to an oligopeptide transporter (OPT; detailed in this report), and Cas95 (accession no. FJ014489), which showed homology to a sugar transporter (92% similarity and 88% identity to *Pyrenophora tritici-repens *EDU43724.1). The Cas51 clone was further characterized.

**Figure 1:**
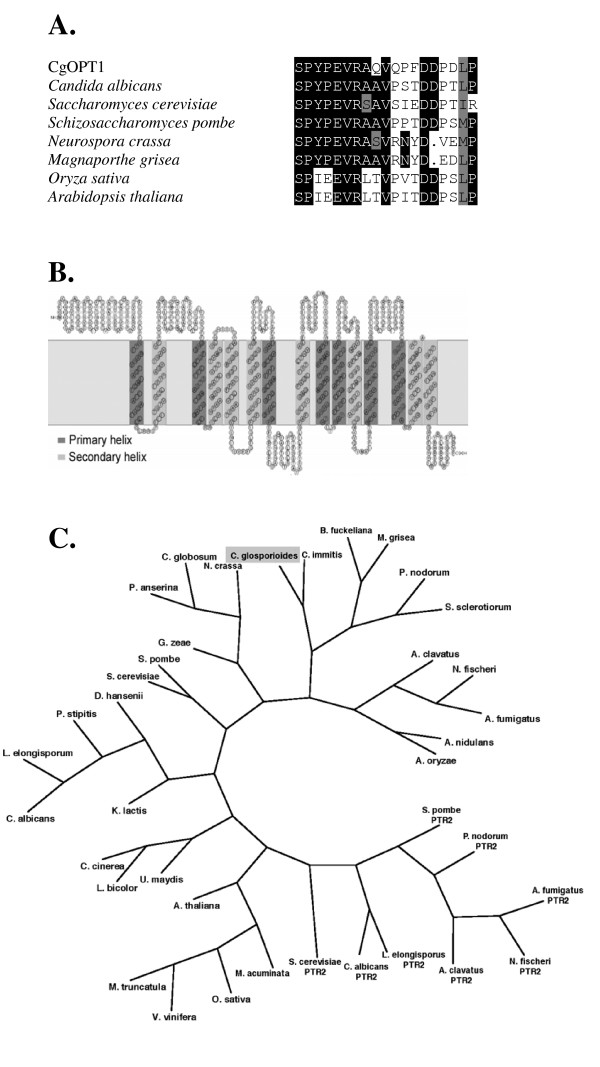
**Sequence analysis of the predicted CgOpt1 protein**. **A**. Multiple alignments (ClustalW) of the CgOpt1 SPYxEVRxxVxxxDDP motif with the motif in OPTs from yeasts, filamentous fungi, and plants. **B**. Topology prediction of the CgOpt1 protein. Transmembrane-domain prediction and topology representation were obtained with SOSUI [30]. Transmembrane domains were also supported by TMHMM analysis [31,32]. **C**. Unrooted phylogenetic tree of fungi and plants with predicted OPTs or OPT-like proteins. *CgOPT1 *sequence is represented by its species name, *C. gloeosporioides*, highlighted in a gray box. Sequences of proteins belonging to the PTR2 peptide transporter family were used as an out group and are termed PTR2. For species names and sequence accession numbers see Additional files 1 and Additional file 2.

The full-length sequence of the Cas51 EST was obtained. BlastX analysis showed strong homology to OPTs from various organisms: *Schizosaccharomyces pombe *Isp4 (accession no. CAC05511.1, 59% similarity and 40% identity), *Aspergillus oryzae *Opt (BAE60512.1, 64% similarity and 48% identity), *Neurospora crassa *Isp4-like (EAA35341.1, 66% similarity and 47% identity), and *Candida albicans *Opt1 (EAK99338.1, 60% similarity and 42% identity). In addition, the Cas51 predicted protein contained 14 transmembrane-spanning domains and the consensus sequence SPYxEVRxxVxxxDDP (Fig. 1A, B), both of which have been found in all previously described OPTs [18].

An unrooted parsimony-based phylogenetic tree grouped Cas51 with OPTs and OPT-like proteins from other fungi (Fig. 1C). OPTs from several other fungi and some plants are grouped in distinct clades, while the other type of peptide transporter (PTR2) is grouped in a separate clade. These analyses clearly demonstrated that the predicted peptide belongs to the OPT family and that it encodes for a putative oligopeptide transporter. The gene was therefore named *CgOPT1 *for *Colletotrichum gloeosporioides *OPT (accession no. FJ008981). The predicted protein contains 752 amino acids, has a predicted mass of 84.9 kDa, and a pI of 8.89. The gene includes three exons separated by two introns of 58 and 73 bp.

### Induction of *CgOPT1 *gene expression by IAA

**Figure 2:**
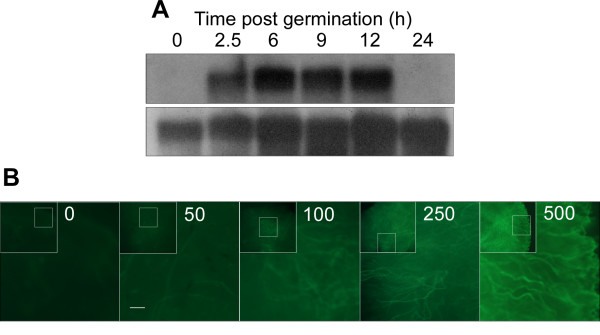
**Activation of *CgOPT1 *transcription by IAA and during spore germination**. **A**. Spores were germinated in pea extract and *CgOPT1 *expression was determined at various time points. Top – *CgOPT1*, bottom – rRNA. **B**. Expression of *CgOPT1 *in mycelia was determined after growing the fungus for 48 h in CD medium (0), CD supplemented with 500 μM tryptophol (Tol), or CD with 100 μM or 500 μM IAA. Top – *CgOPT1*, bottom – rRNA. **C**. The transgenic strain Pop-gfp6 was grown in CD media supplemented with various concentrations of IAA. GFP levels were evaluated 48 h after culture inoculation. Control (0) contained an equal volume of ethanol. Low magnification image is presented as inset in each frame. The portion of the colony that is presented in higher magnification is designated by a small square within each inset. Bars = 20 μm.

Expression of *CgOPT1 *was below detection levels in resting spores, strongly enhanced during spore germination and then reduced again to basal levels during mycelia development (Fig. 2A). Addition of IAA to the mycelia enhanced *CgOPT1 *gene expression, confirming transcriptional activation by auxin (Fig. 2B).

Further expression analyses were performed using a transgenic strain of *C. gloeosporioides*, Popt-gfp6, in which the *GFP *reporter gene is regulated by the *CgOPT1 *promoter. The GFP signal in spores was enhanced during germination with a peak at 12 h and then it decreased, similar to gene-expression results obtained by northern blot analysis (data not shown). To evaluate the response to auxin, the Popt-gfp6-transgenic isolate was grown in Czapek Dox (CD) medium supplemented with IAA and the GFP signal was monitored 48 h after culture inoculation. GFP fluorescence was enhanced by IAA in a concentration-dependent manner, with saturation at 250 μM IAA (Fig. 2C). No change in GFP fluorescence was detected in media supplemented only with ethanol (the solvent used to dissolve IAA).

### Silencing of *CgOPT1 *transcription by RNA interference (RNAi)

**Figure 3:**
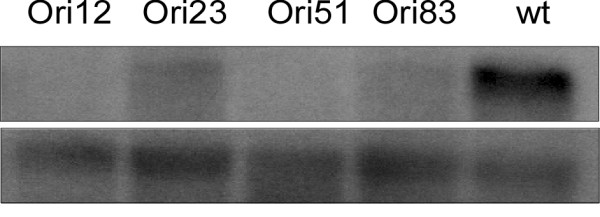
**Silencing of *CgOPT1 *gene expression**. Spores of isolates obtained by transformation with the OptRi (RNAi) plasmid were germinated in pea extract. After 9 h, samples were collected and their RNA extracted. Reduced *CgOPT1 *gene expression is evident in all of the transgenic isolates.

*cgopt1*-silenced mutants were generated and characterized. Because homologous integration does not work well in *C. gloeosporioides *f. sp. *aeschynomene*, mutants were generated by RNA silencing. The wild-type strain was co-transformed with the RNAi cassette OptRi and the gGFP plasmid [19], which was used to confer resistance to hygromycin B. Some of the hygromycin-resistant colonies showed discoloration and reduced sporulation. Spores were collected from culture plates of these isolates and germinated for 9 h in pea extract, conditions under which *CgOPT1 *gene expression is normally high (Fig. 2A). Variable levels of reduced *CgOPT1 *expression were noted in all isolates (Fig. 3). The phenotype of the *cgopt1*-silenced mutants was determined using isolates Ori51 and Ori83.

### Pathogenicity

**Figure 4:**
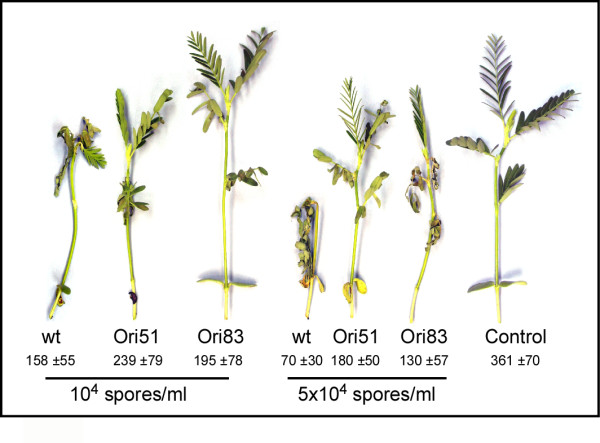
***cgopt1*-silenced mutants exhibit reduced pathogenicity**. *Aeschynomene virginica *plants were inoculated with spore suspensions of wild-type, Ori51 or Ori83 strains. Spores were collected from plates, counted and diluted in water containing 0.05% Tween 20. Plants were sprayed to runoff and then kept in a humid atmosphere for 16 h. Picture was taken 6 days post-inoculation. Numbers are the mean of average fresh weight and SD of six plants. Data from one experiment are presented. Repetition of experiments led to similar results.

Spore-inoculation experiments were performed using several spore dilutions: 10^4^, 5 × 10^4^, and 10^5 ^spores/ml. In all experiments, the *cgopt1*-silenced mutants caused delayed and reduced symptoms compared to wild-type-infected plants (Fig. 4). For example, in a typical experiment using 10^4 ^spores/ml, the wild-type-infected plants developed severe symptoms 5 days post-inoculation, and all six plants died after 8 days, whereas four out of six Ori51- or Ori83-infected plants showed only minor or no symptoms.

### Pigmentation and sporulation

**Figure 5:**
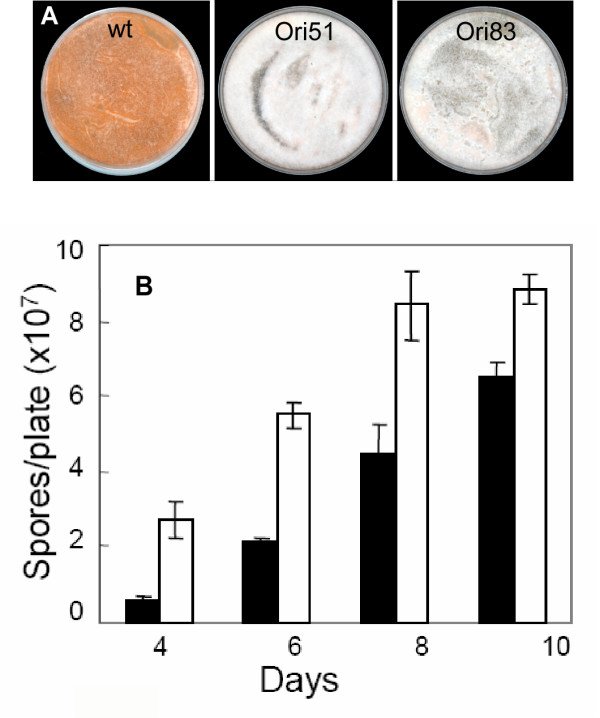
***cgopt1*-silenced mutants exhibit reduced pigmentation and sporulation**. **A**. Wild-type, Ori51 and Ori83 strains were cultured on REG plates. Picture was taken after 8 days. **B**. Sporulation assay: Ori51 (black bars) and wild-type (empty bars) strains were cultured on EMS agar medium in 90-mm Petri dishes. Spores were harvested and counted after 5 days. Data from one experiment are presented. Bars are the mean and SD of five replications. Differences between wild type and the mutant were found significant according to t-test analysis (*P *< 0.05) in each of the time points (days 4, 6, 8, and 10). Repetition of experiments led to similar results.

The *cgopt1*-silenced mutants showed several morphological differences compared to the wild-type strain. When grown on solid regeneration (REG) medium, they produced more aerial hyphae than the wild-type cultures and failed to accumulate the typical orange pigment (Fig. 5A). The mutants also produced fewer spores than the wild type (Fig. 5B). The differences in sporulation between the wild-type and mutant strains were more significant in young cultures and decreased after longer periods of culturing, suggesting delayed sporulation rather than a direct effect on spore formation.

**Figure 6:**
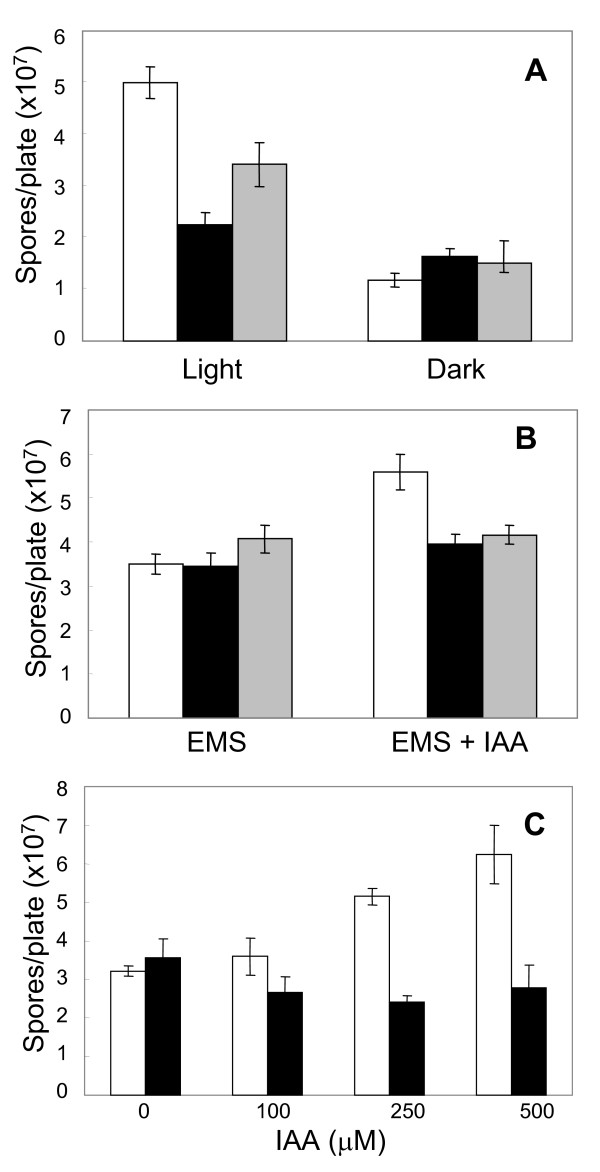
**Effect of IAA on sporulation in wild type and *cgopt1*-silenced mutants**. Strains were cultured on EMS plates with and without IAA. Spores were collected and counted after 5 days. **A**. Plates were kept in continuous light (left) or darkness (right). White bars – wild type, Black bars – Ori51, Gray bars – Ori83. **B**. Fungi were cultured in the dark on EMS (left) or EMS with 500 μM IAA (right). **C**. Fungi were cultured in the dark on EMS plates with increasing IAA concentrations. Bars are the mean and SD of five replications. Differences between wild type and the mutants were found significant according to t-test (*P *< 0.05) in the following treatments: sporulation in the light (A), sporulation in dark on EMS with 500 μM IAA (B, C), sporulation in the dark on EMS with 250 μM (C). Repetition of experiments led to similar results.

To further characterize the sporulation defects in the *cgopt1*-silenced mutants, we compared sporulation in complete darkness: the wild type is known to produce less spores when grown in the dark vs. in the light. Under conditions of complete darkness, the wild type and *cgopt1*-silenced mutants produced similar numbers of spores, lower than the number of spores produced in the light (Fig. 6A). Thus, only light-induced sporulation was affected in the mutants, while sporulation in the dark was unaffected.

Next we tested the possible effect of IAA on sporulation. Wild-type and mutant strains were cultured on media with 500 μM IAA. The plates were kept in the dark to prevent photo-oxidation of IAA, and to eliminate light-induced differences in sporulation between the wild type and mutants. IAA significantly enhanced sporulation in wild-type cultures under these conditions, while it had no effect on sporulation of the *cgopt1*-silenced mutants (Fig. 6B). Furthermore, the effect of IAA on sporulation in wild-type cultures was dose-dependent: a small increase in spore production was observed at 100 μM IAA, and production was further enhanced by 250 μM and 500 μM IAA (Fig. 6C). No change was observed in the sporulation of the mutants, regardless of IAA concentration. These results showed a clear and consistent phenotype caused by IAA, which is abolished in the *cgopt1*-silenced mutants.

### Colony morphology

**Figure 7:**
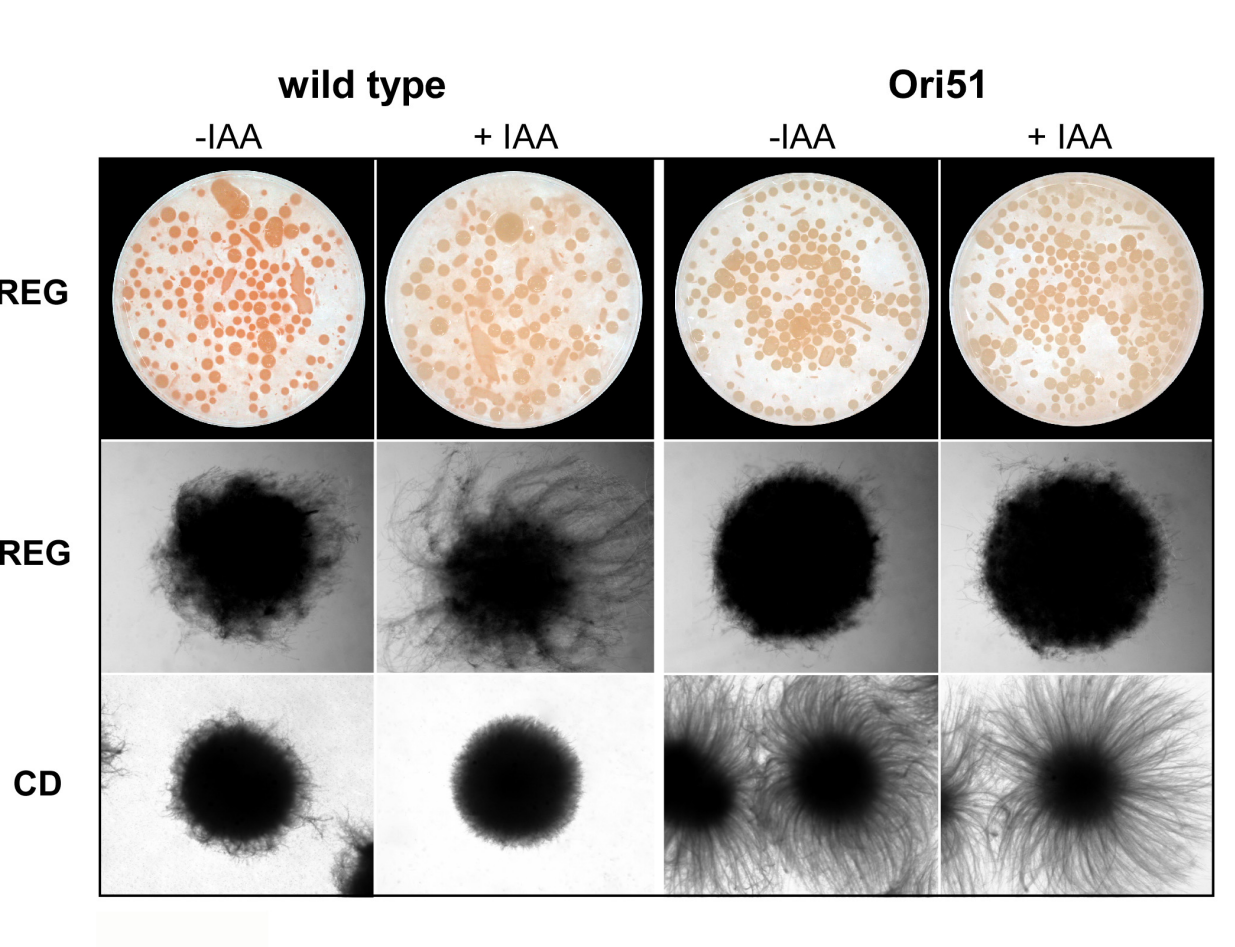
**Effect of culture media and IAA on morphology of wild type and *cgopt1 *mutants**. Similar results were obtained with Ori51 and Ori83 mutant strains. Only the results with Ori51 are presented. Top: Colonies of wild type and Ori51 mutant strain grown in REG liquid medium for 3 days in the absence (-) and presence (+) of 500 μM IAA. Middle: stereoscope images of individual pellets that developed in REG media with or without 500 μM IAA. Bottom: stereoscope images of individual pellets that developed in CD medium with or without 500 μM IAA. Bar = 1 mm.

While characterizing the transcriptional response to IAA, we noticed the development of more compact mycelium in the presence of auxin. To further examine this phenotype, we tested the effect of IAA on the development of mycelia in liquid culture. In REG medium, the wild-type colonies accumulated intense orange pigmentation, while the silenced mutants developed a very pale orange color (Fig. 7, top). This phenotype was similar to that observed on solid REG plates (Fig. 5A). IAA greatly reduced pigmentation in wild-type cultures, whereas it had no effect on the mutants, which retained their light orange color (Fig. 7, top).

A difference was noted in the morphology of wild-type and mutant colonies. In REG medium, the wild type developed pellets surrounded by long hyphae, which became more compact with shorter hyphae when IAA was added (Fig. 7, middle). Under these conditions, the *cgopt1*-silenced mutants developed compact pellets without free hyphae, and this morphology did not change in the presence of IAA. In CD medium, the wild type developed more compact pellets than in REG medium, which became even denser in the presence of IAA (Fig. 7, bottom). The *cgopt1*-silenced mutants developed pellets with very long hyphae (hairy pellets) in CD medium and again, this morphology was not altered by IAA. Thus, the wild-type isolate developed more condensed pellets in IAA-containing media, while the morphology of the *cgopt1*-silenced mutants differed from the wild type, and was unaffected by IAA.

## Discussion

In a previous report, we showed that *C. gloeosporioides *produces auxin both in culture and *in planta *[16,17]. This raised the possibility of auxin involvement in the regulation of fungal development and pathogenicity, and of the existence of auxin-responsive genes regulating fungal responses to IAA. As a first step towards identifying the putative IAA-responsive fungal genes, we constructed a SSH library using mycelia from auxin-containing medium as the tester. Under culture conditions, over 95% of the IAA that is produced by *C. gloeosporioides *is secreted into the medium [20]. We therefore used a relatively high IAA concentration (500 μM), assuming that the endogenous concentrations would be at least 10-fold lower. We also added 500 μM IAM, the intermediate product of IAA production in *C. gloeosporioides *[17]. The SSH yielded limited information on putative IAA-induced genes since only three clones showed consistent induction by IAA. Thus, although putative IAA-induced genes were identified, the results from the SSH approach do not support a massive change in gene transcription by IAA. However, the number of genes that could be tested by SSH was limited and more conclusive results might be obtained through robust transcript analysis using microarrays when such tools become available in *C. gloeosporioides*.

*CgOPT1 *exhibited consistent induction by IAA and was therefore further analyzed. Characterization of the gene as a putative OPT was strongly supported by its overall homology to other OPTs, as well as by the presence of the conserved SPYxEVRxxVxxxDDP sequence and 14 transmembrane domains, which are common to all OPTs [18,21,22]. Further analyses, including complementation of yeast mutants, are needed to determine that CgOPT1 is indeed an oligopeptide transporter and to find substrate specificity.

In *S. cerevisiae*, there are two genetically and physiologically distinct proton-coupled peptide transporter systems: the PTR (peptide transport) and the OPT (oligopeptide transport) protein families. Members of the PTR and OPT families differ in function and they do not share significant sequence homology (see Fig. 1C). PTR proteins are common in all organisms and transport di- or tripeptides. OPT proteins are found only in plants and fungi and transport 4- and 5-amino-acid peptides [22,23]. Metabolically, the transport of small oligopeptides is important as an amino acid, carbon, and nitrogen source [23]. Therefore, silencing of *CgOPT1 *would be expected to reduce the uptake of nutrients from proteinaceous sources, which might have an effect on growth and development. Further, CgOPT1 might facilitate incorporation of metabolites or small peptides that can be used as signalling molecules e.g., during plant infection.

*CgOPT1 *was activated in the presence of IAA in a concentration-dependent manner. Transcription was already enhanced at 50 μM IAA and was further enhanced at higher concentrations, with saturation at 500 μM. These concentrations are much higher than the IAA levels in plants but are within the range of IAA amounts produced by *C. gloeosporioides *[16]. Lack of activation by acetic acid, indole-3-ethanol (tryptophol) or tryptamine ruled out possible activation of *CgOPT1 *by auxin-induced changes in pH, or as a general response to indoles. Nevertheless, at this stage it is impossible to determine whether up-regulation of *CgOPT1 *in the presence of IAA is a direct response to IAA or rather, an indirect response to other changes that might be brought about by IAA. Further, induction by IAA does not necessarily imply that it would be involved in IAA transport, especially because *C. gloeosporioides *produces large quantities of IAA, so induction might be through endogenous rather than exogenous IAA.

In addition to the IAA-induced transcription of *CgOPT1*, the gene was differentially expressed during fungal development, particularly during spore germination. *CgOPT1 *transcript could not be detected in resting spores, it was highly induced during germination, and then it declined during mycelium formation. This expression pattern is opposite to that of the vacuolar copper-transporting gene *CgCTR2*, which is necessary for the initial stages of germination and is highly expressed in resting spores and down-regulated immediately after spore germination [24]. Therefore, CgOpt1 is probably important during germ-tube formation and elongation, but is not required for the initiation of spore germination. Silencing of the gene provided additional evidence for the involvement of CgOptT1 in development as well as pathogenicity: *cgopt1*-silenced mutants displayed reduced sporulation and pigmentation, and were less pathogenic than the wild-type strain. These pleiotropic effects suggest association of CgOpt1 with several different processes.

IAA appears to have an enhancing effect on processes such as sporulation, spore germination, and germ-tube elongation. However, the effects of IAA vary with experimental conditions, and opposite results might be obtained. In our sporulation assay, we took special care to eliminate possible interference and side effects from experimental parameters such as solvent, medium, or light. IAA was applied to filter paper, the ethanol was evaporated, and then the filter was placed between two layers of agar to avoid direct contact with the fungus. Additionally, because sporulation is enhanced by light, the experiments were conducted under both light and dark conditions. Under both conditions, we observed consistent and significant enhancement of sporulation in the wild type by IAA (Fig. 6). This procedure therefore provided a reliable assay for the determination of responses to IAA in the wild type and *cgopt1*-silenced mutants.

The *cgopt1*-silenced mutants exhibited reduced sporulation compared to the wild type when grown in the light. This difference was not observed in the dark, where both the wild type and mutants produced reduced, but equal numbers of spores (Fig. 6A). Thus, CgOpt1 is probably associated only with light-dependent sporulation, and is not required for light-independent sporulation. However, IAA had no effect on sporulation in the mutants, unlike the significant enhancement of sporulation observed in the wild-type strain. These results suggest that IAA and light enhance sporulation through different pathways, and that CgOpt1 is associated with the IAA-dependent pathway, but not the light-dependent one. In addition, morphological differences were observed between the wild type and *cgopt1 *mutants when grown in liquid culture, and the addition of IAA induced morphological changes in the wild type, but had almost no effect on the mutants (Fig. 7). Thus both sporulation and pellet morphology, which differ between the wild type and *cgopt1*-silenced mutants, are affected by IAA in the wild type but not in the *cgopt1 *mutants. These results suggest that CgOpt1 might be associated with developmental pathways that are also affected by IAA. The abolishment of a response to IAA in the *cgopt1 *mutants is surprising and further research is needed to determine the connection between *CgOPT1 *and IAA.

## Conclusion

Although fungi are capable of producing IAA, its purpose, if any, is unclear. Here we present evidence that IAA promotes sporulation and causes changes in growth morphology in the fungal plant pathogen *C. gloeosporioides*. These results suggest the importance of IAA to fungal development and reproduction. In addition, we identified an IAA-responsive gene which appears to be involved in mediating IAA's effects. At this stage however, the underlying mechanism is unknown and further investigation is needed.

## Methods

### Fungi

The following media were used: regeneration (REG) medium (per liter): 145 g mannitol, 4 g yeast extract, 1 g soluble starch, 16 g agar; Czapek Dox (CD) medium (per liter): 3 g NaNO_3_, 0.5 g MgSO_4_·7H_2_O, 0.5 g KCl, 55 mg FeSO_4_, 30 g sucrose, 1 g KH_2_PO_4_; Emerson's YpSs (EMS) medium (per liter): 4 g yeast extract, 2.5 g soluble starch, 1 g K_2_HPO_4_, 0.5 g MgSo_4_; pea extract: 900 g of frozen peas boiled in 1.6 liters of water and then filtered. All solid media contained 18 g agar and were supplemented with 100 mg/ml chloramphenicol. Fungi were cultured under continuous fluorescent light as previously described [25]. For liquid cultures, 50 ml medium was inoculated with 10^7 ^spores that were collected from a 5-day-old colony. The flasks were placed on a rotary shaker (180 rpm) and incubated at 28°C. Three separate flasks were used for each treatment, and the experiments were repeated at least three times.

### Construction of SSH library

Spores of *C. gloeosporioides *were collected from 5-day-old culture plates and germinated in pea extract for 4 h. Germinated spores were washed once with sterile water and then transferred to 250-ml flasks containing 50 ml CD medium or CD supplemented with 500 μM each of IAM and IAA (Sigma-Aldrich). The flasks were incubated with agitation for 24 h after which the mycelium was collected and its RNA extracted. Total RNA and mRNA were extracted using Sigma GenElute mammalian total RNA miniprep kit and GenElute mRNA miniprep kit, respectively. The PCR-Select cDNA subtraction kit (Clontech) was used to produce an SSH library containing putative IAA-induced clones. The final PCR products were cloned into pTZ57R vector (Fermentas). Single colonies were collected and PCR was performed on 76 colonies using the nested 1 (5'-TCGAGCGGCCGCCCGGGCAGGT-3'), nested 2R (5'-AGCGTGGTCGCGGCCGAGGTAAA-3') primers from the PCR-Select cDNA subtraction kit. Thirty-five clones were sequenced resulting in 24 different ORFs. DNA of the corresponding ESTs was amplified by PCR, separated on a 1% agarose gel, blotted onto a Hybond-N^+ ^membrane (Amersham) and hybridized with ^32^P-labeled cDNA probes that were generated from IAA-exposed [(+) probe] and IAA-unexposed [(-) probe] mycelium. Clones that differentially hybridized only with the (+) probe were analyzed by northern blot hybridization.

### Northern blot analysis

Total RNA (2 to 5 μg) was used for northern blot analysis. Samples were separated on a formaldehyde denaturing 1% agarose gel and blotted onto a Hybond-N^+ ^membrane. DNA fragments of *C. gloeosporioides *ribosomal 18s gene were amplified by PCR from *C. gloeosporioides *genomic DNA using the primer 5'CGGAGAAGGAGCCTGAG/GGCCCAAGGTTCAACTACGAG-3'. cDNA probes were radiolabeled with ^32^P-dCTP and hybridized to the membranes according to standard protocols.

### Isolation of *CgOPT1*

*CgOPT1 *genomic DNA was isolated using the Universal Genome Walker kit (Clontech). Two rounds of PCR were performed using ExTaq enzyme (TaKaRa), first with primer CAS 51-GW-rev (5'-CTCGTAGACGAAAGTACTGGCACC-3') and then with primer CAS 51-GW-rev2 (5'-TCGTCGAAGGGTTGGACCTGCGC-3'). PCR products obtained by this procedure were cloned into the pTZ57R A/T cloning vector and sequenced.

### Plasmid construction

Plasmid Popt-gfp was constructed for expression of GFP under control of the *CgOPT1 *promoter. A 1.5-kb region upstream of the *CgOPT1 *start codon was amplified by PCR, introducing a 5' *Bgl*II restriction site and a 3' *Nco*I restriction site. The fragment was inserted into a gGFP plasmid at *Bgl*II/*Nco*I, replacing the *gpd *promoter upstream of *GFP *[19]. Popt-gfp was co-transformed into *C. gloeosporioides *together with the pAN701 plasmid which carries the hygromycin-resistance cassette. Plasmid OptRi was constructed for RNAi-mediated silencing of *CgOPT1*. Two DNA fragments corresponding to bases 1100–2000 (up-Ri) and 1100–1700 (down-Ri) of the *CgOPT1 *gene were amplified by PCR and cloned into vector pTZ57. The up-Ri fragment was cloned into the *Sph*I/*Spe*I site of the pTZ57-down-Ri plasmid. The plasmid was cut with *Bam*HI/*Bgl*II and the fragment was cloned into *Bam*HI of vector ksgt between the *gpd *promoter and *TrpC *terminator, resulting in plasmid OptRi. The OptRi plasmid was transformed into *C. gloeosporioides *together with the gGFP vector, which confers resistance to hygromycin.

### Fungal transformation

Fungal transformation was performed by electroporation of germinated spores as previously described [20]. Hygromycin-resistant colonies were collected and the presence of either Popt-gfp or OptRi plasmid was verified by PCR. Transgenic isolates obtained with the Popt-gfp plasmid were compared and detailed analyses were performed with isolate Popt-gfp6. For OptRi, isolates containing the silencing cassette were propagated and the expression levels of *CgOPT1 *were compared. Detailed analyses were carried out with isolates Ori51 and Ori83, which gave similar results in all cases.

### Sporulation assay

Fungi were cultured on CD or EMS plates. For media with IAA, the calculated amount of IAA was dissolved in ethanol and applied on a Whatman filter paper, the ethanol was air-dried and then the filter was placed between two layers of agar medium. Plates were prepared 1 day before inoculation to allow diffusion of IAA into the medium. Control plates were prepared in a similar fashion with filters containing an equivalent volume of air-dried ethanol. Each plate was inoculated with a 3-mm^2 ^mycelium cube that was excised from a 5-day-old culture. After 5 days, the spores were washed from the plates and counted. Three plates were used as replicates in each experiment and all experiments were repeated several times. Data are the mean results of three experiments.

### Plant inoculation

Inoculation experiments were performed with 12-day-old *Aeschynomene virginica *plants as described previously [26]. Plants were sprayed to runoff with spore suspension containing 0.05% (v/v) Tween 20. Control plants were sprayed with similar volumes of 0.05% Tween 20. Six plants per treatment were used as replicates in each experiment and all experiments were repeated several times. Symptoms were recorded and fresh weight determined 6 days post-inoculation.

### Microscopy

Fluorescent and light microscopy were performed with a Zeiss Axioskop 2 epifluorescent microscope, or with an Olympus SZX 12 fluorescent stereoscope equipped with an eGFP filter. Confocal microscopy was performed with a Zeiss CLSM 510 laser-scanning confocal microscope.

### Computational analysis

*CgOPT1 *homologous sequences were identified by BlastpX [27] analyses at the NCBI database http://www.ncbi.nlm.nih.gov/. For details of species and retrieved sequences see Additional file 1 and Additional file 2. Multiple alignments were performed by the ClustalW program [28]. Phylogenetic analyses were conducted with the PHYLIP package [29], available online at http://mobyle.pasteur.fr. The phylogenetic tree was based on a parsimony analysis using the Protpars program and subsequently drawn with the Drawtree program.

Data were analyzed by t-test at a significance level of *P *< 0.05, using the Microsoft Office Excel software package.

### Accession number

The GenBank accession number for the *CgOPT1 *gene analyzed in this study is FJ008981.

## List of abbreviations

IAA: Indole-3-acetic acid; IPY: Indole-3-pyruvic acid; IAM: Indole-3-acetamide; SSH: Suppressive subtraction hybridization; OPT: Oligopeptide transporters; CD: Czapek Dox; REG: Regeneration medium; PTR: Peptide Transport; EMS: Emerson's YpSs medium; PE: Pea extract; Tol: Tryptophol.

## Authors' contributions

VC carried out planning and execution of experiments related to figures 5, 6, 7, participated in preparation of figure 1 and was involved in writing of manuscript. RM carried out experiments related to figures 2, 3 and 4, was involved in experiments presented in figure 1. AS conceived the study, and participated in its design and coordination. All authors read and approved the final manuscript.

## Supplementary Material

Additional file 1 **Sequences used for phylogenetic analysis**. Homology of CgOPT1 to related sequences from other fungi is presented. When opt is quoted, the sequence is referenced as OPT1 member in the database. Blast results are the output of blastp analyses done with the translated sequence of CgOpt1. Click here for file

Additional file 2 **PTR2 sequences used for phylogenetic analysis**. Accession numbers of PTR2 sequences that were used for phylogenetic analysis are presented. Click here for file

## References

[B1] Tudzynski B Sharon A Osiewacz HD Biosynthesis, biological role and application of fungal phytohormones The Mycota, Vol. X Industrial Applications 2001 Berlin, Sprnger-Verlag 183 211

[B2] Ek M Ljunquist PO Stenstrom E Indole-3-acetic acid production by mycorrhizal fungi determined by Gas Chromatography-Mass Spectrometry New Phytol 1983 94 401 407 10.1111/j.1469-8137.1983.tb03454.x

[B3] Furukawa T Koga J Adachi T Kishi K Syono K Efficient conversion of L-tryptophan to indole-3-acetic acid and/or tryptophol by some species of *Rhizoctonia* Plant Cell Physiol 1996 37 899 905

[B4] Ona O Van Impe J Prinsen E Vanderleyden J Growth and indole-3-acetic acid biosynthesis of *Azospirillum brasilense *Sp245 is environmentally controlled FEMS Microbiol Lett 2005 246 125 132 10.1016/j.femsle.2005.03.048 15869971

[B5] Sosa-Morales ME Guevara-Lara F Martinez-Juarez VM Parades-Lopez O Production of indole-3-acetic acid by mutant strains of *Ustilago maydis *(maize smut/huitlacoche) App Microbiol Biotechnol 1997 48 726 729 10.1007/s002530051123

[B6] Kamisaka S Yanagishima N Masuda Y Effect of auxin and gibberellin on sporulation in yeast Physiol Plant 1967 20 90 97 10.1111/j.1399-3054.1967.tb07145.x

[B7] Prusty R Grisafi P Fink GR The plant hormone indole acetic acid induces invasive growth in *Saccharomyces cerevisiae* Proc Natl Acad Sci USA 2004 101 4153 4157 10.1073/pnas.0400659101 15010530 PMC384710

[B8] Nakamura T Tomita K Kawanabe Y Murayama T Effect of auxin and gibberellin on spore germination in *Neurospora crassa *II. "Spore density effect" and auxin Plant Cell Physiol 1982 23 1363 1369

[B9] Eckert SE Hoffmann B Wanke C Braus GH Sexual development of *Aspergillus nidulans *in tryptophan auxotrophic strains Arch Microbiol 1999 172 157 166 10.1007/s002030050755 10460886

[B10] Tsavkelova EA Klimova YS Cherdyntseva TA Netrusov AI Microbial producers of plant growth stimulators and their practical use: A review App Biochem Microbiol 2006 42 133 143 16761564

[B11] Barash I Manulis-Sasson S Recent evolution of bacterial pathogens: the gall-forming *pantoea agglomerans *case Annu Rev Phytopathol 2009 47 13352 10.1146/annurev-phyto-080508-081803 19400643

[B12] Le Floch G Rey P Benizri E Benhamou N Tirilly Y Impact of auxin-compounds produced by the antagonistic fungus *Pythium oligandrum *or the minor pathogen *Pythium *group F on plant growth Plant Soil 2003 257 459 470 10.1023/A:1027330024834

[B13] Terrile MC Olivieri FP Bottini R Casalongue CA Indole-3-acetic acid attenuates the fungal lesions in infected potato tubers Physiol Plant 2006 127 205 211 10.1111/j.1399-3054.2006.00667.x

[B14] Laurans F Pepin R Gay G Fungal auxin overproduction affects the anatomy of *Hebeloma cylindrosporum *– *Pinus pinaster *ectomycorrhizae Tree Physiol 2001 21 533 540 11359711 10.1093/treephys/21.8.533

[B15] Cohen B Amsellem Z Maor R Sharon A Gressel J Transgenically-enhanced expression of IAA confers hypervirulence to plant pathogens Phytopathology 2002 92 590 596 10.1094/PHYTO.2002.92.6.590 18944254

[B16] Reineke G Heinze B Schirawski J Buttner H Kahmann R Basse CW Indole-3-acetic acid (IAA) biosynthesis in the smut fungus *Ustilago maydis *and its relevance for increased IAA levels in infected tissue and host tumor formation Mol Plant Pathol 2008 9 339 355 10.1111/j.1364-3703.2008.00470.x 18705875 PMC6640242

[B17] Robinson M Riov J Sharon A Indole-3-acetic acid biosynthesis in *Colletotrichum gloeosporioides *f. sp. *aeschynomene* App Environ Microbiol 1998 64 5030 5032 10.1128/aem.64.12.5030-5032.1998 PMC90963 9835603

[B18] Maor R Haskin S Kedmi-Levi H Sharon A Biosynthesis, regulation and *in planta *auxin production by *Colletotrichum gloeosporioides *f. sp. *aeschynomene* App Environ Microbiol 2004 69 1695 1701 10.1128/AEM.70.3.1852-1854.2004 PMC368304 15006816

[B19] Lubkowitz MA Barnes D Breslav M Burchfield A Naider F Becker JM *Schizosaccharomyces pombe isp4 *encodes a transporter representing a novel family of oligopeptide transporters. Mol Microbiol Mol Microbiol 1998 28 429 741 10.1046/j.1365-2958.1998.00827.x 9643541

[B20] Maor R Puyesky M Horwitz BA Sharon A Use of green fluorescent protein (GFP) for studying development and fungal-plant interaction in *Cochliobolus heterostrophus* Mycol Res 1998 102 491 496 10.1017/S0953756297005789

[B21] Robinson M Sharon A Transformation of the bioherbicide *Colletotrichum gloeosporioides *f. sp. *aeschynomene *by electroporation of germinated spores Curr Genet 1999 36 98 104 10.1007/s002940050478 10447601

[B22] Koh S Wiles AM Sharp JS Naider FR Becker JM Stacey G An oligopeptide transporter gene family in *Arabidopsis* Plant Physiol 2002 128 21 29 10.1104/pp.010332 11788749 PMC148940

[B23] Lubkowitz MA Hauser L Breslav M Naider F Becker JM An oligopeptide transport gene from *Candida albicans* Microbiology 1997 143 387 396 10.1099/00221287-143-2-387 9043116

[B24] Hauser M Narita V Donhardt AM Neider F Becker JM Multiplicity and regulation of genes encoding peptide transporters in *Saccharomyces cerevisiae* Mol Mem Biol 2001 18 105 112 11396605

[B25] Barhoom S Kupiec M Xu J-R Sharon A Functional characterization of CgCTR2, a vacuole copper transporter that is necessary for germination and pathogenicity in *Colletotrichum gloeosporioides* Eukar Cell 2008 7 1098 1108 10.1128/EC.00109-07 PMC2446676 18456860

[B26] Barhoom S Sharon A cAMP regulation of pathogenic and saprophytic fungal spore germination Fung Genet Biol 2004 41 317 326 10.1016/j.fgb.2003.11.011 14761792

[B27] Barhoom S Sharon A Bcl-2 proteins link programmed cell death with growth and morphogenetic adaptations in the fungal plant pathogen *Colletotrichum gloeosporioides*. Fung Genet Biol Fung Genet Biol 2007 44 32 43 10.1016/j.fgb.2006.06.007 16950636

[B28] Altschul SF Madden TL Schäffer AA Zhang J Zhang Z Miller W Lipman DJ Gapped BLAST and PSI-BLAST: a new generation of protein database search programs Nuc Acid Res 1997 25 3389 3402 10.1093/nar/25.17.3389 PMC146917 9254694

[B29] Thompson JD Gibson TJ Plewniak F Jeanmougin F Higgins DG The ClustalX windows interface: flexible strategies for multiple sequence alignment aided by quality analysis tools Nuc Acid Res 1997 24 4876 4882 10.1093/nar/25.24.4876 PMC147148 9396791

[B30] Felsenstein J PHYLIP Phylogeny Inference Package Cladistics 1989 5 164 166

[B31] Hirokawa T Boon-Chieng S Mitaku S SOSUI: classification and secondary structure prediction system for membrane proteins Bioinformatics 1998 14 378 379 10.1093/bioinformatics/14.4.378 9632836

[B32] Krogh A Larsson B von Heijne Sonnhammer ELL Predicting transmembrane protein topology with a hidden Markov model: Application to complete genomes J Mol Biol 2001 305 567 580 10.1006/jmbi.2000.4315 11152613

