# Supplementary material for: CgOpt1, a putative oligopeptide transporter from Colletotrichum gloeosporioides that is involved in responses to auxin and pathogenicity
Source: BMC Microbiol. 2009 Aug 21;9:173. doi: 10.1186/1471-2180-9-173 (PMC2769210; doi:10.1186/1471-2180-9-173)
Supplement: Additional file 1 — Sequences used for phylogenetic analysis. Homology of CgOPT1 to related sequences from other fungi is presented. When opt is quoted, the sequence is referenced as OPT1 member in the database. Blast results are the output of blastp analyses done with the translated sequence of CgOpt1. [file 1471-2180-9-173-S1.doc]

# Additional files

| **Taxonomy** | **Species** | **Note** | Acc. # | E value | % identity | %Similarity |
| --- | --- | --- | --- | --- | --- | --- |
| Ascomycota | *Aspergillus fumigatus* | opt | EAL93821.1 | 0.0 | 47 | 63 |
|  | *Aspergillus nidulans* |  | EAA62177.1 | 0.0 | 45 | 62 |
|  | *Aspergillus clavatus* | opt | EAW14385.1 | 0.0 | 49 | 64 |
|  | *Aspergillus oryzae* | opt | BAE60512.1 | 0.0 | 48 | 64 |
|  | *Botryotinia fuckeliana* |  | EDN20268.1 | 1e-167 | 41 | 60 |
|  | *Candida albicans* | opt | EAK99338.1 | 6e-170 | 42 | 60 |
|  | *Chaetomium globosum* |  | EAQ89699.1 | 0.0 | 47 | 66 |
|  | *Coccidioides immitis* |  | EAS34503.1 | 0.0 | 42 | 63 |
|  | *Debaryomyces hansenii* |  | CAG86501.1 | 1e-173 | 44 | 64 |
|  | *Gibberella zeae* |  | FG08669.1 | 0.0 | 49 | 67 |
|  | *Kluyveromyces lactis* |  | CAG99050.1 | 2e-169 | 42 | 63 |
|  | *Lodderomyces elongisporus* | opt | EDK41887.1 | 5e-162 | 41 | 61 |
|  | *Magnaporthe grisea* |  | EDK05381.1 | 2e-180 | 46 | 66 |
|  | *Neosartorya fischeri* | opt | EAW19091.1 | 0.0 | 46 | 64 |
|  | *Neurospora crassa* | opt | EAA35341.1 | 0.0 | 47 | 66 |
|  | *Phaeosphaeria nodorum* |  | EAT89962.2 | 2e-165 | 40 | 60 |
|  | *Pichia stipitis* |  | ABN68276.2 | 5e-170 | 44 | 63 |
|  | *Podospora anserina* |  | CAP64894.1 | 0.0 | 47 | 67 |
|  | *Saccharomyces cerevisiae* |  | P40897 | 3e-126 | 34 | 55 |
|  | *Sclerotinia sclerotiorum* |  | EDN92492.1 | 0.0 | 46 | 64 |
|  | *Schizosaccharomyces pombe* | opt | CAC05511.1 | 3e-164 | 40 | 59 |
| Basidiomycota | *Coprinopsis cinerea* |  | EAU83423.1 | 2e-161 | 41 | 61 |
|  | *Laccaria bicolor* | opt | EDR06085.1 | 5e-161 | 40 | 59 |
|  | *Ustilago maydis* |  | EAK85622.1 | 2e-148 | 39 | 57 |
| Plants | *Arabidopsis thaliana* | opt | Q9FG72 | 3e-125 | 37 | 53 |
|  | *Medicago trunculata* |  | ABN05713.1 | 3e-127 | 37 | 56 |
|  | *Oryza sativa* | opt | BAB89477.1 | 1e-127 | 37 | 55 |
|  | *Vitis vinifera* |  | CAO61570.1 | 2e-129 | 38 | 55 |
|  | *Musa acuminata* | opt | ABF70152.1 | 6e-122 | 37 | 55 |

Supplementary table S1: Sequences used for phylogenetic analysis. Note: when opt is quoted, the sequence is referenced as OPT1 member in the database. Blast results are the output of blastp analyses done with the translated sequence of CgOpt1.
