# Supplementary material for: CgOpt1, a putative oligopeptide transporter from Colletotrichum gloeosporioides that is involved in responses to auxin and pathogenicity
Source: BMC Microbiol. 2009 Aug 21;9:173. doi: 10.1186/1471-2180-9-173 (PMC2769210; doi:10.1186/1471-2180-9-173)
Supplement: Additional file 2 — PTR2 sequences used for phylogenetic analysis. Accession numbers of PTR2 sequences that were used for phylogenetic analysis are presented. [file 1471-2180-9-173-S2.doc]

Supplementary table S2: PTR2 sequences used for phylogenetic analysis.

| **Species** | Accession number |
| --- | --- |
| Aspergillus clavatus | EAW10971.1 |
| *Aspergillus fumigatus* | EAL84745.1 |
| *Candida albicans* | EAK93530.1 |
| Lodderomyces elongisporus | EDK44067.1 |
| Neosartorya fischeri | EAW20888.1 |
| *Phaeosphaeria nodorum* | AAO31597.1 |
| *Saccharomyces cerevisiae* | CAA82172.1 |
| *Schizosaccharomyces pombe* | CAB99397.1 |
